# The Use of Feed Additives to Reduce the Effects of Aflatoxin and Deoxynivalenol on Pig Growth, Organ Health and Immune Status during Chronic Exposure 

**DOI:** 10.3390/toxins5071261

**Published:** 2013-07-17

**Authors:** Alexandra C. Weaver, M. Todd See, Jeff A. Hansen, Yong B. Kim, Anna L. P. De Souza, Tina F. Middleton, Sung Woo Kim

**Affiliations:** 1 Department of Animal Science, North Carolina State University, Raleigh, NC 27695, USA; E-Mails: acchayto@ncsu.edu (A.C.W.); todd_see@ncsu.edu (M.T.S.); jeffhansen@murphybrownllc.com (J.A.H.); 2 Murphy-Brown LLC, Rose Hill, NC 28458, USA; E-Mail: anadesouza@murphybrownllc.com; 3 Department of Population Health & Pathobiology, North Carolina State University, Raleigh, NC 27695, USA; E-Mail: youngbaek_kim@ncsu.edu; 4 Ag ProVision LLC, Kenansville, NC 28349, USA; E-Mail: tmiddle@aol.com

**Keywords:** aflatoxin, clays, deoxynivalenol, pigs, yeast

## Abstract

Three feed additives were tested to improve the growth and health of pigs chronically challenged with aflatoxin (AF) and deoxynivalenol (DON). Gilts (*n* = 225, 8.8 ± 0.4 kg) were allotted to five treatments: CON (uncontaminated control); MT (contaminated with 150 µg/kg AF and 1100 µg/kg DON); A (MT + a clay additive); B (MT + a clay and dried yeast additive); and C (MT + a clay and yeast culture additive). Average daily gain (ADG) and feed intake (ADFI) were recorded for 42 days, blood collected for immune analysis and tissue samples to measure damage. Feeding mycotoxins tended to decrease ADG and altered the immune system through a tendency to increase monocytes and immunoglobulins. Mycotoxins caused tissue damage in the form of liver bile ductule hyperplasia and karyomegaly. The additives in diets A and B reduced mycotoxin effects on the immune system and the liver and showed some ability to improve growth. The diet C additive played a role in reducing liver damage. Collectively, we conclude that AF and DON can be harmful to the growth and health of pigs consuming mycotoxins chronically. The selected feed additives improved pig health and may play a role in pig growth.

## 1. Introduction

Mycotoxins are toxic secondary metabolites of fungi commonly found on grains, which can cause severe impacts on animal health and performance [1]. Of 300 to 400 known mycotoxins, aflatoxin (AF) and deoxynivalenol (DON) are two of the most common and harmful for the feed and animal industries [2]. It is estimated that 25% of the world’s crops are contaminated to some extent by mycotoxins, and a survey from the NC Cooperative Extension Service found that 17% of corn tested was contaminated with levels higher than 20 µg/kg AF, and over 60% of corn contained DON [3,4,5]. For both toxins, swine are one of the most sensitive species. As a result of the high contamination of corn by these mycotoxins, pigs may easily consume grains contaminated with AF or DON above the USA FDA action level of 20 µg/kg to 200 µg/kg AF and advisory level of 1000 µg/kg DON for pigs [6]. When consumed, these mycotoxins can decrease pig growth, cause immune dysfunction or damage organs [7,8,9,10]. Although the fungi producing AF and DON grow under different environmental conditions, making it unlikely that they contaminate grains at the same time, there is, however, a high likelihood that two grain sources contaminated with AF or DON can be mixed together during feed processing [1]. As a result, pigs may consume a diet co-contaminated with mycotoxins. Together, these mycotoxin may act synergistically to further the negative effects caused to animals [11,12].

One method to reduce the detrimental impact of mycotoxin contamination is the use of feed additives. Clays and yeast components may have the ability to reduce the impact of mycotoxins through binding properties [5,13]. Efficacy of these feed additives can depend on the types of mycotoxins and their concentrations in feed. One type of clay includes montmorillonite, a layered silicate with properties that allows adsorption of the polar AF compound by the exchange of cations [13,14]. Previous research has documented that 1% montmorillonite was able to adsorb 98% to 99.5% of AF during an intestinal fluid simulation [14]. Other clays include sodium bentonite and sepiolite. Sodium bentonite is a layered crystalline structured clay formed from volcanic ash and may consist primarily of montmorillonite [15]. Sodium bentonite is often used as a binding agent in the production of pelleted foods. Sepiolite is a silicate clay also commonly used as a pellet binder. Both of these clays have been shown to adsorb nearly all of the existing AF when testing in simulated intestinal fluid [14]. Diatomaceous earth is another material that may be used in the animal health industry. Diatomaceous earth is a sedimentary mineral formed from the skeletal remains of a group of algae called diatoms and contains silica, sodium, magnesium, iron and other trace minerals [16]. Due to its properties, diatomaceous earth is commonly used as an anti-caking agent during feed processing. 

Yeasts may also be beneficial during a mycotoxin challenge. Dried brewer’s yeast is dried to preserve a portion of the fermenting and adsorptive power of the yeast [17]. This material has mycotoxin absorption abilities, because the yeast cell walls can contain polysaccharides, lipids and proteins with absorption centers that bind mycotoxins through hydrogen and ionic bonding or hydrophobic interactions [13]. Yeast cell wall materials have a particular ability to bind mycotoxins, such as DON, zearalenone and ochratoxin [15]. Yeast culture is a second type of yeast additive that may be used to reduce mycotoxin effects. Yeast culture is a dried, fermented product containing small amounts of live yeast and the metabolic products produced by the yeast during fermentation [18]. Yeast culture can act primarily as a probiotic to stimulate the immune system and to improve gut health, in turn reducing mycotoxin effects. It is hypothesized for this current study that pigs eating diets naturally contaminated with AF and DON together will have reduced weight gain, an altered immune system and increased organ damage, whereas the use of clay and yeast feed additives will reduce these negative effects. The objective of this study was to determine the effects of AF and DON together from naturally contaminated grains on growth, organ health and immune responses of pigs and to investigate the efficacy of three uniquely different types of feed additives to reduce these effects during this mycotoxin challenge.

## 2. Results

### 2.1. Growth Performance

The body weight of pigs did not differ between treatments for all trial weeks, nor did average daily gain (ADG) for the first three weeks (Table 1). During the third week of the trial, feeding mycotoxins tended to lower ADG, but this did not continue during the last two weeks of the trial. However, over the entire 42-day period, pigs consuming mycotoxins tended to have reduced ADG in contrast to pigs not consuming AF and DON. Daily feed intake was not altered by the mycotoxins when considering the entire trial period, and gain-to-feed ratio was also not affected. The addition of the feed additives did not significantly improve the growth of pigs consuming mycotoxins, although the feed additives in diet A and B did numerically improve performance. 

**Table 1:** Performance of pigs fed diets containing mycotoxins and various feed additives.

	Treatment ^1^					SEM	*p*-Value
	CON	MT	A	B	C		
BW, kg							
day 0	8.9	8.9	8.8	8.9	8.9	0.1	0.987
day 7	9.7	9.8	9.6	9.9	9.8	0.2	0.880
day 14	11.5	11.6	11.5	11.6	11.7	0.3	0.987
day 21	13.9	13.9	13.5	14.0	14.0	0.4	0.877
day 28	17.6	16.7	17.2	17.0	17.2	0.4	0.733
day 35	21.6	20.2	20.6	20.7	20.3	0.5	0.311
day 42	26.6	24.8	25.6	25.5	25.0	0.5	0.124
ADG, g/d							
day 0 to 7	114	120	113	144	128	26	0.909
day 7 to 14	261	261	274	245	281	24	0.855
day 14 to 21	347	328	281	337	319	30	0.567
day 21 to 28	518 ^a^	401 ^b^	523 ^a^	429 ^a,b^	457 ^a,b^	36	0.073
day 28 to 35	572	494	496	534	453	36	0.183
day 35 to 42	724	655	705	674	670	24	0.273
day 0 to 42	423 ^a^	377 ^b^	399 ^a,b^	394 ^a,b^	385 ^b^	12	0.079
ADFI, g/d							
day 0 to 7	326	308	306	322	317	20	0.948
day 7 to 14	545	546	541	531	553	29	0.988
day 14 to 21	730 ^a^	622 ^b^	643 ^b^	637 ^b^	630 ^b^	30	0.088
day 21 to 28	909	838	885	837	866	38	0.634
day 28 to 35	994	948	1024	980	936	47	0.691
day 35 to 42	1392	1288	1349	1272	1279	47	0.303
day 0 to 42	816	759	791	763	764	27	0.518
Gain/feed							
day 0 to 7	0.210	0.349	0.186	0.332	0.285	0.101	0.724
day 7 to 14	0.478	0.477	0.510	0.391	0.484	0.052	0.569
day 14 to 21	0.484	0.529	0.432	0.535	0.520	0.046	0.487
day 21 to 28	0.612	0.471	0.603	0.512	0.537	0.057	0.367
day 28 to 35	0.633 ^a^	0.512 ^b^	0.477 ^b^	0.543 ^a,b^	0.469 ^b^	0.045	0.083
day 35 to 42	0.531	0.516	0.531	0.537	0.527	0.023	0.975
day 0 to 42	0.524	0.495	0.507	0.518	0.502	0.012	0.469

^a,b^ Means within a row with different superscripts show a tendency to differ (0.05 ≤ *p* < 0.10); ^1^ CON: uncontaminated control; MT: contaminated with 150 µg/kg AF and 1100 µg/kg deoxynivalenol (DON); A: MT + 2 mg/kg of a clay additive; B: MT + 1.5 mg/kg of a clay and dried yeast additive; C: MT + 1.1 mg/kg of a clay and yeast culture additive. BW, body weight; ADG, average daily gain; ADFI, average daily feed intake.

### 2.2. Hematological Measurements

Hematological analysis on day 28 showed that some changes did occur, due to the mycotoxins (Table 2). Pigs consuming mycotoxins alone had higher blood levels of mean corpuscular hemoglobin (MCH) than pigs not consuming mycotoxins. These pigs also tended to have a higher mean corpuscular hemoglobin concentration (MCHC) and had increased cellular mean corpuscular volume (MCV) than pig consuming the diet without mycotoxins. The three feed additives, in particular, the additive in diet B, significantly reduced the effects of AF and DON on MCH and MCHC. By day 42, pigs consuming mycotoxins had significantly increased hematocrit and a continued increase in MCH and MCV in contrast to pigs not fed mycotoxins (Table 3). These pigs also had an increased monocyte count. The consumption of the additive in diet A lowered the levels of MCV and monocytes in the blood, but both additives A and C actually increased blood concentrations of MCHC, while the additive in C increased basophil concentration. Pigs fed the additive in diet B had significantly lower MCV and monocyte counts without affecting other hematological parameters in the pigs.

**Table 2:** Hematology (day 28) of pigs fed diets containing mycotoxins and various feed additives.

	Treatment ^1^						
	CON	MT	A	B	C	SEM	*p-*Value
Hematocrit, %	32.9	35.1	39.6	38.6	36.3	2.4	0.421
Hemoglobin, g/dL	10.3	12.0	12.7	12.3	11.6	0.8	0.297
MCH ^2^, pg	15.9 ^a^	16.8 ^c^	16.8 ^c^	16.1 ^a,b^	16.6 ^b,c^	0.2	0.009
MCHC ^2^, g/dL	32.1 ^d^	32.6 ^e^	32.0 ^d^	32.0 ^d^	32.1 ^d^	0.2	0.098
MCV ^2^, fL	49.7 ^a^	51.5 ^b,c^	52.5 ^c^	50.5 ^a,b^	51.7 ^b,c^	0.6	0.018
Platelet, 10^3^/µL	382	415	359	378	298	39	0.277
RBC ^2^, 10^6^/µL	6.63	6.82	7.52	7.58	7.01	0.43	0.423
WBC ^2^, 10^3^/µL	26.2	24.1	26.3	24.7	21.8	1.5	0.236
Basophil, 10^3^/µL	0.23	0.16	0.32	0.31	0.19	0.06	0.209
Eosinophil, 10^3^/µL	0.58	0.55	0.58	0.47	0.44	0.09	0.730
Lymphocyte, 10^3^/µL	11.4	10.5	12.5	11.3	10.4	0.9	0.458
Monocyte, 10^3^/µL	1.70	2.04	2.04	1.73	1.58	0.18	0.221
Neutrophil, 10^3^/µL	11.8	10.4	10.2	10.2	8.6	1.0	0.265

^a–c ^Means within a row with different superscripts differ (*p* < 0.05); ^d,e ^Means within a row with different superscripts show a tendency to differ (0.05 ≤ *p* < 0.10); ^1^ CON: uncontaminated control; MT: contaminated with 150 µg/kg AF and 1100 µg/kg DON; A: MT + 2 mg/kg of a clay additive; B: MT + 1.5 mg/kg of a clay and dried yeast additive; C: MT + 1.1 mg/kg of a clay and yeast culture additive; ^2 ^MCH: mean corpuscular hemoglobin; MCHC: mean corpuscular hemoglobin concentration; MCV: mean corpuscular volume; RBC: red blood cell count; WBC: white blood cell count.

**Table 3:** Hematology (day 42) of pigs fed diets containing mycotoxins and various feed additives.

	Treatment ^1^								
	CON	MT	A	B	C	SEM	*p*-Value
Hematocrit, %	34.7 ^a^	37.5 ^b^	36.5 ^ab^	34.9 ^a^	35.4 ^a^	0.9	0.029
Hemoglobin, g/dL	10.9	11.5	11.6	10.9	11.2	0.3	0.172
MCH ^2^, pg	16.9 ^a^	17.8 ^b,c^	17.8 ^b,c^	17.2 ^a,b^	18.1 ^c^	0.3	0.008
MCHC ^2^, g/dL	31.4 ^d,e^	30.7 ^d^	31.8 ^e^	31.2 ^d,e^	31.9 ^e^	0.4	0.072
MCV ^2^, fL	53.9 ^a^	58.1 ^c^	56.0 ^b^	55.3 ^a,b^	56.8 ^b,c^	0.7	0.001
Platelet, 10^3^/µL	431	410	370	329	369	28	0.101
RBC ^2^, 10^6^/µL	6.44	6.46	6.47	6.32	6.24	0.15	0.622
WBC ^2^, 10^3^/µL	18.6	21.4	19.6	18.0	20.1	1.4	0.386
Basophil, 10^3^/µL	0.09 ^d^	0.16 ^d,e^	0.12 ^d,e^	0.13 ^d,e^	0.20 ^e^	0.03	0.084
Eosinophil, 10^3^/µL	0.61	0.57	0.83	0.46	0.60	0.13	0.404
Lymphocyte, 10^3^/µL	9.56	9.18	10.10	8.83	9.49	0.53	0.513
Monocyte, 10^3^/µL	0.97 ^a^	1.43 ^b^	1.05 ^a^	0.95 ^a^	1.26 ^a,b^	0.13	0.043
Neutrophil, 10^3^/µL	7.39	10.05	7.53	7.60	8.40	1.17	0.362

^a–c ^Means within a row with different superscripts differ (*p* < 0.05); ^d,e ^Means within a row with different superscripts tend to differ (0.05 ≤ *p* < 0.10); ^1^ CON: uncontaminated control; MT: contaminated with 150 µg/kg AF and 1100 µg/kg DON; A: MT + 2 mg/kg of a clay additive; B: MT + 1.5 mg/kg of a clay and dried yeast additive; C: MT + 1.1 mg/kg of a clay and yeast culture additive; ^2 ^MCH: mean corpuscular hemoglobin; MCHC: mean corpuscular hemoglobin concentration; MCV: mean corpuscular volume; RBC: red blood cell count; WBC: white blood cell count.

### 2.3. Immunological Evaluation

On day 28, immunological parameters were minimally altered. However, pigs fed the additive in diet B tended to have an increased serum immunoglobulin (Ig) G concentration in contrast to pigs consuming only mycotoxins or mycotoxins plus the additive in diet A (Table 4). On day 42, pigs consuming AF and DON tended to have increased IgG and IgM compared with pigs consuming the CON or A diets, whereas serum concentrations of IgG and IgM in pigs, the other additives in B and C were intermediate to the mycotoxin contaminated and uncontaminated controls. The cytokine tumor necrosis factor alpha (TNFα) was not affected by the mycotoxins or the feed additives throughout the trial.

**Table 4:** Immunological parameters of pigs fed diets containing mycotoxins and various feed additives.

	Treatment ^1^					SEM	*p*-Value
	CON	MT	A	B	C		
Day 28							
IgG ^2^, mg/mL	25.3 ^a,b^	18.0 ^b^	20.0 ^b^	28.7 ^a^	18.7 ^a,b^	1.5	0.096
IgM ^2^, mg/mL	2.46	2.69	2.54	2.86	2.70	0.12	0.937
TNFα ^2^, pg/mL	131	115	164	141	143	16	0.142
Day 42							
IgG, mg/mL	10.5 ^a,b^	15.1 ^c^	8.6 ^a^	13.5 ^b,c^	11.1 ^a,b,c^	0.6	0.069
IgM, mg/mL	3.02 ^a^	4.39 ^b^	2.71 ^a^	3.19 ^a,b^	3.23 ^a,b^	0.15	0.077
TNFα, pg/mL	118	97	108	132	118	38	0.519

^a–c ^Means within a row with different superscripts tend to differ (0.05 ≤ *p* < 0.10); ^1^ PCON: uncontaminated control; MT: contaminated with 150 µg/kg AF and 1,100 µg/kg DON; A: MT + 2 mg/kg of a clay additive; B: MT + 1.5 mg/kg of a clay and dried yeast additive; C: MT + 1.1 mg/kg of a clay and yeast culture additive; ^2^ IgG: immunoglobulin G; IgM: immunoglobulin M; TNFα: tumor necrosis factor α.

### 2.4. Liver Biochemistry

Liver biochemical parameters measured in the serum were minimally affected by mycotoxin consumption. On day 28, the serum albumin concentration of pigs fed MT was increased, and the additives in diets B and C numerically reduced this concentration (Table 5). Mycotoxin consumption without feed additives increased BUN to creatinine ratio, which was reduced by feeding of additive B, and pigs fed MT also had increased serum nitrogen. On day 42, albumin continued to be elevated due to the mycotoxins, and serum calcium was also increased (Table 6). Generally, the addition of the feed additives did not alter the biochemical parameters affected by the mycotoxins.

**Table 5:** Serum biochemistry (day 28) of pigs fed diets containing mycotoxins and various feed additives.

	Treatment ^1^					SEM	*p*-Value
	CON	MT	A	B	C		
Albumin, g/dL	2.12 ^a^	2.39 ^b^	2.43 ^b^	2.17 ^a,b^	2.29 ^a,b^	0.08	0.043
Albumin:globulin ^2^	0.62 ^a^	0.70 ^a,b^	0.77 ^b^	0.59 ^a^	0.71 ^a,b^	0.05	0.038
Alk phos ^2^, U/L	227	232	240	205	262	19	0.165
ALT ^2^, U/L	27.1	29.9	30.3	27.5	30.1	2.7	0.866
AST ^2^, U/L	32.9	41.7	44.0	39.9	38.3	3.7	0.307
Bilirubin, mg/L	10.8	10.7	10.0	10.0	10.0	0.4	0.526
BUN:creatinine ^2^	20.5 ^a^	25.2 ^c^	24.6 ^b,c^	23.9 ^b^	27.4 ^c^	1.2	0.006
Calcium, mg/dL	9.92	10.16	10.29	9.79	9.88	0.16	0.129
Chloride, mEq/L	98.8	98.7	99.2	99.2	99.5	0.5	0.777
Cholesterol, mg/dL	77.4	79.2	74.8	74.5	76.6	3.2	0.783
CPK ^2^, U/L	527	437	768	544	405	125	0.246
Creatinine, mg/dL	0.65	0.63	0.64	0.67	0.63	0.02	0.383
Globulin, g/dL	3.60 ^d,e^	3.51 ^d,e^	3.32 ^d^	3.80 ^e^	3.34 ^d^	0.16	0.098
Glucose, mg/dL	94	91	98	94	100	4	0.687
Na:K ^2^	23.9	23.8	22.5	22.9	22.9	0.7	0.260
Nitrogen, mg/dL	13.2 ^a^	15.5 ^b^	15.5 ^b^	15.7 ^b^	16.8 ^b^	0.6	0.003
Phosphorus, mg/dL	9.02	9.55	9.80	9.29	9.75	0.24	0.130
Potassium, mEq/L	5.98	6.05	6.35	6.28	6.18	0.19	0.420
Protein, g/dL	5.72	5.90	5.75	5.97	5.63	0.13	0.222
Sodium, mEq/L	141	141	141	142	141	1	0.975

^a–c ^Means within a row with different superscripts differ (*p* < 0.05); ^d,e ^Means within a row with different superscripts tend to differ (0.05 ≤ *p* < 0.10); ^1^ CON: uncontaminated control; MT: contaminated with 150 µg/kg AF and 1,100 µg/kg DON; A: MT + 2 mg/kg of a clay additive; B: MT + 1.5 mg/kg of a clay and dried yeast additive; C: MT + 1.1 mg/kg of a clay and yeast culture additive; ^2 ^Albumin:globulin: albumin to globulin ratio; Alk phos: alkaline phosphatase; ALT: alanine aminotransferase; AST: aspartate aminotransferase; BUN:creatinine: BUN (blood urea nitrogen) to creatinine ratio: CPK: creatine phosphokinase; Na:K: sodium to potassium ratio.

**Table 6:** Serum biochemistry (day 42) of pigs fed diets containing mycotoxins and various feed additives.

	Treatment^1^					SEM	*p*-Value
	CON	MT	A	B	C		
Albumin, g/dL	2.49 ^a^	2.80 ^b^	2.94 ^b^	2.63 ^a,b^	2.76 ^a,b^	0.10	0.025
Albumin:globulin ^2^	0.81 ^d^	0.93 ^d,e^	0.98 ^e^	0.79 ^d^	0.96 ^d,e^	0.06	0.095
Alk phos ^2^, U/L	233	236	234	209	250	15	0.350
ALT ^2^, U/L	27.1	29.5	29.3	29.3	32.6	1.9	0.391
AST ^2^, U/L	31.5	38.8	36.7	46.9	42.3	4.3	0.125
Bilirubin, mg/L	10.0	10.0	10.0	10.0	10.7	0.3	0.473
BUN:creatinine ^2^	16.0 ^d^	20.6 ^d,e^	21.9 ^e^	20.8 ^d,e^	23.0 ^e^	1.0	0.078
Calcium, mg/dL	9.87 ^a^	10.29 ^b,c^	10.53 ^c^	9.93 ^a^	10.12 ^a,b^	0.13	0.002
Chloride, mEq/L	101	101	102	101	102	1	0.576
Cholesterol, mg/dL	78.7	76.9	72.8	72.6	75.2	2.4	0.389
CPK ^2^, U/L	656	723	548	636	715	177	0.473
Creatinine, mg/dL	0.66	0.64	0.64	0.68	0.65	0.02	0.551
Globulin, g/dL	3.25	3.17	3.05	3.37	3.00	0.14	0.367
Glucose, mg/dL	111	110	114	105	108	3	0.378
Na:K ^2^	26.0	25.3	24.9	26.0	25.3	0.6	0.825
Nitrogen, mg/dL	12.3 ^a^	13.0 ^a,b^	13.9 ^b,c^	13.7 ^b,c^	14.6 ^c^	0.5	0.018
Phosphorus, mg/dL	8.95	9.63	9.51	9.02	9.37	0.24	0.171
Potassium, mEq/L	5.57	5.73	5.82	5.55	5.75	0.14	0.772
Protein, g/dL	5.74	5.97	5.99	5.99	5.76	0.12	0.248
Sodium, mEq/L	143	143	144	143	143	1	0.641

^a–c ^Means within a row with different superscripts differ (*p* < 0.05); ^d,e ^Means within a row with different superscripts tend to differ (0.05 ≤ *p* < 0.10); ^1^ CON: uncontaminated control; MT: contaminated with 150 µg/kg AF and 1,100 µg/kg DON; A: MT + 2 mg/kg of a clay additive; B: MT + 1.5 mg/kg of a clay and dried yeast additive; C: MT + 1.1 mg/kg of a clay and yeast culture additive; ^2 ^Albumin:globulin: albumin to globulin ratio; Alk phos: alkaline phosphatase; ALT: alanine aminotransferase; AST: aspartate aminotransferase; BUN:creatinine: BUN to creatinine ratio: CPK: creatine phosphokinase; Na:K: sodium to potassium ratio.

### 2.5. Weight, Color and Histology of Internal Organs

Both liver weight and liver weight as a percent of body weight were increased in pigs fed the mycotoxins, whereas the additive in diet B reduced this effect, so that weights were similar to those in the uncontaminated control (Table 7). Kidney and spleen weights were not different between treatments. Minolta color measurements of lightness (L*) and redness (a*) for the liver were not altered, but liver yellowness (b*) tended to be increased in pigs fed feed additives. Kidney and spleen color characteristics were not different among treatments. 

**Table 7:** Tissue weight and color of pigs fed diets containing mycotoxins and various feed additives.

	Treatment ^1^					SEM	*p*-Value
	CON	MT	A	B	C		
Organ weight, g							
Liver	719 ^a^	800 ^b^	840 ^b^	714 ^a^	778 ^a,b^	30	0.015
Kidney	149	146	159	141	147	7	0.432
Spleen	61.1	58.5	65.0	56.4	57.7	4.1	0.578
Organs,% of body weight							
Liver	2.71 ^a^	3.29 ^b^	3.31 ^b^	2.80 ^a^	3.14 ^b^	0.10	0.001
Kidney	0.56	0.60	0.62	0.55	0.59	0.02	0.131
Spleen	0.23	0.24	0.25	0.22	0.24	0.01	0.583
Color liver ^2^							
Lightness (L*)	34.7	35.6	35.2	35.2	35.6	0.5	0.729
Redness (a*)	14.0	13.8	13.8	13.7	13.9	0.2	0.904
Yellowness (b*)	3.17 ^a^	3.60 ^a,b^	3.90 ^a,b^	3.94 ^a,b^	4.65 ^b^	0.37	0.083
Color kidney							
Lightness (L*)	45.5	45.9	46.3	47.0	45.8	0.7	0.610
Redness (a*)	13.2	13.5	13.1	12.3	13.4	0.6	0.550
Yellowness (b*)	7.69	7.34	7.76	8.88	8.29	0.46	0.144
Color spleen							
Lightness (L*)	35.7	35.9	36.4	37.0	36.0	0.7	0.756
Redness (a*)	18.1	18.4	18.1	18.3	18.4	0.3	0.847
Yellowness (b*)	1.89	1.87	1.79	1.75	1.71	0.21	0.970

^a,b ^Means within a row with different superscripts differ (*p* < 0.05); ^c,d ^Means within a row with different superscripts tend to differ (0.05 ≤ *p* < 0.10); ^1^ CON: uncontaminated control; MT: contaminated with 150 µg/kg AF and 1,100 µg/kg DON; A: MT + 2 mg/kg of a clay additive; B: MT + 1.5 mg/kg of a clay and dried yeast additive; C: MT + 1.1 mg/kg of a clay and yeast culture additive; ^2^ Tissue color measured via Minolta Colorimeter (Konica Minolta, Ramsey, NJ).

**Table 8:** Percent damage to tissues of pigs fed diets containing mycotoxins and various feed additives.

	Treatment ^1^					SEM	*p*-Value
	CON	MT	A	B	C		
Liver ^2^							
Bile ductule hyperplasia	2.6 ^b^	5.3 ^a^	3.9 ^a,b^	3.0 ^b^	4.0 ^a,b^	0.6	0.014
Fibrosis	3.9	3.5	4.9	3.9	4.0	0.6	0.627
Hydropic degeneration	8.1 ^a,b^	9.5 ^a^	6.3 ^b^	9.1 ^a^	8.5 ^a^	0.7	0.021
Inflammation	2.4	3.0	2.6	3.0	2.8	0.2	0.219
Karyomegaly	2.8 ^c^	29.3 ^a^	11.1 ^b^	5.8b ^c^	5.5 ^b,c^	2.8	0.001
Necrosis	2.2	2.8	2.6	3.0	2.8	0.2	0.206
Vacuolation	3.9	6.7	5.8	4.4	5.5	0.8	0.130
Kidney ^2^							
Fibrosis	2.6	3.0	2.8	2.8	2.9	0.3	0.944
Necrosis	2.8	2.8	2.8	2.8	2.8	0.2	0.935
Protein casts	2.6	2.8	2.8	2.6	2.6	0.3	0.922
Regeneration	2.4	3.0	2.6	2.8	2.4	0.3	0.387
Vacuolation	2.6 ^e^	3.9 ^d^	3.5 ^d,e^	3.0 ^d,e^	2.4 ^e^	0.4	0.065

^a–c ^Means within a row with different superscripts differ (*p* < 0.05); ^d,e ^Means within a row with different superscripts show a tendency to differ (0.05 ≤ *p* < 0.10); ^1^ CON: uncontaminated control; MT: contaminated with 150 µg/kg AF and 1100 µg/kg DON; A: MT + 2 mg/kg of a clay additive; B: MT + 1.5 mg/kg of a clay and dried yeast additive; C: MT + 1.1 mg/kg of a clay and yeast culture additive; ^2 ^Microscopic examinations indicating percent of damage to tissue: normal to minimal (0% to 5%); mild (5% to 15%); moderate (15% to 40%); severe (greater than 40%).

Tissue damage occurred by the feeding of the mycotoxins (Table 8). Pigs fed AF and DON had increased hepatic bile ductile hyperplasia, karyomegaly and hydropic degeneration. The additive in diet B had the strongest ability to reduce the effects of the mycotoxins on the liver. However, the other feed additives also reduced karyomegaly. Vacuolation was the only form of kidney damage observed due to the mycotoxins, and in this case, the additive in diet C reduced this damage. All other measurements of liver and kidney damage were not different between pigs fed MT, CON and the feed additives.

## 3. Discussion

Aflatoxin and DON are mycotoxins that commonly contaminate North American grains, such as corn, wheat and barley [1,15]. When ingested by swine, these mycotoxins can reduce growth and feed intake, challenge the immune system and cause organ damage [7,10,19,20]. These mycotoxin effects on pig health can result in significant economic losses for producers. When AF and DON are combined in the diet, they may cause further negative impacts on the animal than when consumed alone. Previous research indicates that these mycotoxin may act synergistically to cause greater negative effects on animals [11,12]. Although the design of the present study did not allow for differentiation of the effects of AF and DON alone or together, these results do provide valuable information on how AF and DON alter pig growth and health when mixed together during feed processing. As a result, this study provides practical insight on how pigs may be affected by mycotoxins under normal feeding practices. 

There are many commercially available feed additives with the potential to reduce the toxicity of mycotoxins [4,13,14]. Some materials, such as clays, are currently used as anti-caking agents to improve flow during feed processing, but their secondary role may be to serve as mycotoxin binding agents [15,21]. The current research aimed to determine the ability of three uniquely different types of feed additives to ameliorate the chronic negative effects of feeding diets containing 150 µg/kg AF and 1100 µg/kg DON to pigs for 42 days. The feed additive in diet A consisted of montmorillonite clay, whereas the feed additive in diet B was comprised of the clays, sodium bentonite and sepiolite, and also contained a dried brewer’s yeast component. Finally, diet C contained a mixture of diatomaceous earth and yeast culture. Each of these clay and yeast materials may have the ability to reduce the effects of mycotoxins on pigs. 

In this study, the impact of including feed additives in the diets without mycotoxins was not investigated, as the researchers were trying only to determine how the three additives differed in their effects on pigs during a mycotoxin challenge. The impact of the feed additives when consumed in uncontaminated diets have been shown previously. Generally, clay additives do not appear to impact the animal either positively or negatively when consumed without mycotoxins. Research conducted by [22] showed that feeding montmorillonite clay to weanling pigs did not alter growth performance. The additions of the clays sodium bentonite, sepiolite or diatomaceous earth have also not been shown to be beneficial or detrimental to performance of swine or poultry when added to a diet uncontaminated by mycotoxins [16,23,24]. In mice, clay additives are shown to cause an increase in serum potassium concentrations, potentially due to binding reactions, but this effect is not yet duplicated in pigs or poultry [25]. Supplementing yeast cell material to pig diets not contaminated with mycotoxins can result in variable effects. In some cases, yeast supplementation can increase ADG and ADFI, alter feed-to-gain ratio or enhance the immune system and protect against bacterial infections [26,27,28,29]. However, other studies show that supplementation of live yeast or yeast β-glucan does not alter growth performance or provide an immune modulating effect to weanling pigs [28,30,31]. In our current study, we are unable to provide details on the effects of the additives alone, as the design of this study did not include the additives with the uncontaminated feed. However, the aim of our research was not to determine the specific binding properties of these additives, but rather, to determine how these additives impacted pigs when consumed simultaneously during a mycotoxin challenge. 

The consumption of AF and DON by pigs has been shown to decrease ADG at both low and high concentrations [10,32,33,34]. Data from [35] showed that 300 µg/kg AF and 600 µg/kg DON can cause 5% reductions in the growth of pigs. Over the entire trial period in our study, pigs fed mycotoxins had a 10.8% decrease in ADG. This result is lower than [10], where pigs fed mycotoxin concentrations of 180 µg/kg AF in combination with 900 µg/kg DON had reduced ADG by 21%. However, AF levels were lower (150 µg/kg) and DON higher (1100 µg/kg) in our present study than the experiment by [10], which may indicate a stronger effect of AF on growth than DON. Other studies found AF and DON effects on ADG comparable to our current study [8,19,36,37]. The addition of three feed additives to the contaminated diet resulted in a numerical increase in ADG, but these values were not significantly different than feeding of AF and DON alone. 

Feed intake is shown to decrease when feeding pigs low AF concentrations, as indicated by [33], where feed intake of pigs was reduced 3.5%, due to 140 µg/kg AF. Deoxynivalenol alone is not shown to decrease ADFI at a low concentration of 280 µg/kg [38]. However, [10] showed that low AF and DON together (180 µg/kg AF, 900 µg/kg DON) tended to reduce ADFI by 15.4% over a 33 day period (*p* = 0.061). Our current study showed a minimal effect on ADFI. However, the three feed additives did result in a numerical improvement in feed intake, although not significant. Gain-to-feed ratio was generally unaffected, as well, and other research agrees with our findings [10,33,39,40]. 

Based on these results, it has been determined that at chronic low levels, AF (150 µg/kg) and DON (1100 µg/kg) resulted in minimal effects on growth performance. Although not significantly improving growth parameters, the three feed additives showed potential benefits for reducing mycotoxin effects. Other studies indicate that the clay feed additives can improve the ADG and ADFI of pigs fed 200 to 800 µg/kg AF [36,37,41]. In contrast, [34] indicated that yeast cell wall material did not improve ADG, ADFI or gain-to-feed ratio. As a result of the previous and current research, it appears that the ability of feed additives to reduce mycotoxin affects is variable, and their function may depend on other factors, such as mycotoxin type, contamination level and pig health status. 

To determine how mycotoxins act within the body, hematological, immunological and biochemical variables were determined. Hematological analysis showed a few differences between treatments. The primary effects occurred for MCH, MCHC, MCV, hematocrit and monocyte counts. There were some variations in hematology between day 28 and 42; however, more effects were observed as mycotoxin consumption time increased. Although not abnormal levels for pigs of this age [42,43], the hematological differences found for pigs fed mycotoxins do show a general effect on red blood cells (RBC). The measurement of MCHC is the average concentration of hemoglobin in a given volume of RBCs, and MCV represents the average volume of a RBC [44]. These measurements can be important for classification of anemia and RBC disorders. In the current study, pigs fed mycotoxins had increased MCH and MCV, indicating that the mycotoxins caused a slight increase in RBC volume. Despite the results observed in this current study, previous research has shown no effect on MCH, MCHC or MCV when pigs are fed 280 to 3000 µg/kg DON [10,38,45]. Hematocrit, which is measured as the concentration of RBC in a given volume of blood and is related to dehydration [44,46,47,48], was also higher in pigs fed mycotoxins. This result suggests that pigs consuming AF and DON may have been dehydrated. This effect is confirmed by [49], where increased hematocrit was observed in pigs fed 2200 to 2500 µg/kg DON, but feeding AF alone at 200 µg/kg did not affect hematocrit [37]. Monocytes, a subset of white blood cells (WBCs), were also significantly increased by the mycotoxins. Previous research has found monocytes to be increased in pigs fed high concentrations of DON [49].

The three feed additives had variable effects on the hematological parameters, although the feed additive in treatment B showed the strongest ability to maintain hematological values closest to the uncontaminated control. However, the addition of both A and B feed additives reduced monocyte levels, which were increased by the mycotoxins. Other research has shown that clays may be beneficial in reducing effects on hematological parameters by AF and DON [15]. Yeast cell materials are also indicated to prevent an increase in monocytes after feeding of AF and DON [34]. 

Proper function of the immune system is important for growing pigs. The adaptive immune system provides a specific immune response, which includes the production of antibodies, such as IgG and IgM, by B-lymphocytes against a particular pathogen or foreign substance [50]. This adaptive immune response is developed over the lifetime of an individual as an adaptation to infection. In our current study, immunological analysis showed minimal treatment effects on day 28, but tendencies were observed on day 42, where IgG and IgM were increased in pigs fed the mycotoxins. These results indicated that low levels of AF and DON stimulated B-lymphocytes to produce more antibodies. Previously, low levels of 140 to 280 µg/kg AF or 280 to 900 µg/kg DON showed no change in IgG or IgM [10,33,38]. On the other hand, high levels of 2200 to 6800 µg/kg DON have been shown to increase concentrations of IgA and IgM, but with no effect on IgG [8,34,49,51]. Previous research has shown yeast materials to be beneficial at preventing an increase in IgG and IgM, due to AF, DON and ZEA (zearalenone) contamination [19,34]. Generally, the addition of the three feed additives to the mycotoxin contaminated diet in our study resulted in immunoglobulin values similar to those in pigs consuming uncontaminated feed. However, the montmorillonite clay product in A showed the strongest ability to reduce immunoglobulin levels. Since clays have a stronger ability to bind AF, these results may indicate that AF had a stronger effect on the immune system of these pigs than DON. On both day 28 and 42, the cytokine TNFα was not altered by the mycotoxin consumption. This cytokine was analyzed as a measure of systemic inflammation, occurring due to the mycotoxins, indicating a pro-inflammatory action of the immune system [52]. Despite a lack of significant difference observed in the current study, previous research does indicate that AF and DON can increase TNFα concentrations in pigs [10]. 

Similar to the hematological analysis, liver biochemistry showed minimal effects of the mycotoxins and the feed additives. At the end of the study, effects occurred primarily in albumin, calcium and nitrogen levels in the serum. Albumin, which is a major protein synthesized by the liver [53], was increased by mycotoxin consumption. However, as mentioned previously, pigs fed the mycotoxins also showed increased hematocrit concentration, which is the most common cause of increased albumin, due to a concentrating effect in the blood following dehydration [47]. Previous research with mycotoxins shows inconsistencies, where albumin may be increased, decreased or unaltered at both low and high AF and DON consumption [8,10,20,54]. The effect on serum calcium was similar to albumin, where mycotoxins were fed alone. The three feed additives did not greatly benefit the pigs in regard to the liver biochemistry. However, it is questioned as to how strongly the liver biochemistry was affected by the mycotoxins, as changes may have been simply due to a dehydration effect rather than the mycotoxins. Further research is needed to determine why the mycotoxins caused changes to the serum biochemistry and if this result is harmful to pigs. 

Organ weights were comparable with data found in previous research after feeding pigs ranges of 50 to 1807 µg/kg AF and 750 to 6510 µg/kg DON [9,10,45,55]. In our current study, liver weight as a percent of body weight was increased in pigs consuming mycotoxins, whereas the feed additive in diet B helped to reduce this effect on the liver. Similar results are shown by [39], where 3000 µg/kg AF increased liver and spleen weights as a percent of body weight. Organ colors were minimally altered; however, tissue damages were observed. It is previously documented that 1000 µg/kg DON causes organ damage, including necrosis, blood vessel thickening and hemorrhage [19,20]. High AF (3000 µg/kg) in the diet is shown to cause liver lesions and vacuolation with portal fibrosis and bile duct hyperplasia [39]. In a previous study by [10], liver fibrosis was the only tissue damage observed when feeding pigs low concentrations of AF and DON. Liver damages were prevalent in our current study for pigs fed the mycotoxin contaminated diet, with increases in hepatic bile ductile hyperplasia and karyomegaly, an increase in nuclear size of a cell [56]. The feed additive in diet B, with clay and dried yeast, was able to significantly reduce both the bile ductile hyperplasia and karyomegaly, whereas the additives in A and C reduced only karyomegaly. Mild hydropic degeneration was observed in all treatments, but was lowest in A, indicating that this form of liver damage may have occurred, due to an unknown factor other than the mycotoxin effect, and the clay product in A helped to reduce this damage. Previous research has shown the benefits of montmorillonite clay to reduce multi-organ toxicities in swine diets containing 1300 µg/kg zearalenone [57]; however, our current research shows that the additive in B composed of clay and dried yeast may also be helpful at reducing damages to organs during a mycotoxin challenge. 

## 4. Experimental Section

### 4.1. Animals*,* Design and Diets

Two hundred twenty five gilts (8.8 ± 0.4 kg, crossbred pigs, Smithfield Premium Genetics, Rose Hill, NC, USA) were used in this study. They were housed in solid concrete floor indoor pens (1.42 m × 3.86 m) at the North Carolina Swine Evaluation Station (Clayton, NC, USA). Pigs were randomly assigned to five dietary treatments within 15 weight blocks. Each pen had three pigs. 

Corn naturally contaminated with AF (270 µg/kg) and barley naturally contaminated with DON (30,000 µg/kg) were used to make a negative control diet with 150 µg/kg AF and 1100 µg/kg DON (Table 9). This diet was derived from [10], who demonstrated that similar concentrations of AF and DON resulted in at least a 15% reduction in growth performance and changes in hematological, biochemical, histological and immune parameters. Three feed additives composed of clay and yeast materials were added to the mycotoxin contaminated diet. To determine mycotoxin contamination levels, 10 samples from different bin locations were taken for each ingredient and finished diet [58,59,60]. The 10 samples were then thoroughly blended together, and two subsamples were collected for determination of mycotoxin content. Analysis of the concentrations of aflatoxin, DON and other mycotoxin contamination in the grains and diet samples was conducted by North Dakota State Veterinary Diagnostic Laboratory (Fargo, ND, USA). Concentrations of AF were determined using an Agilent’s 1100 series HPLC system with a limit of quantitation of 20 µg/kg. The level of DON in contaminated grains was determined using gas chromatography-mass spectrometry with a quantitation limit of 500 µg/kg. Non-contaminated corn and barley were also used in order to formulate a diet without mycotoxins. 

Pigs were fed experimental diets based on their assigned treatment groups: CON (uncontaminated control); MT (contaminated with 150 µg/kg AF and 1100 µg/kg DON); A (MT + a clay additive, 2 mg/kg); B (MT + a clay and dried yeast additive, 1.5 mg/kg); and C (MT + a clay and yeast culture additive, 1.1 mg/kg). Levels of additives in the diets were based on the amount recommended by each manufacturer. Diet A contained an additive composed of a processed calcium montmorillonite clay (Calibrin^®^-A Enterosorbent, Amlan International, Chicago, IL, USA). Calibrin^®^-A montmorillonite contains: 70% to 78% silicon dioxide, 11% to 15% aluminum trioxide, 4% to 7% ferric oxide, 2% to 3% magnesium oxide, 1% to 2% potassium oxide and trace amounts of calcium oxide, sodium oxide and other trace metals. The diet B additive (Unike, Nutriad International NV, Belgium) was composed of sodium bentonite and sepiolite clays along with brewer’s dried yeast. The additive in diet C (no commercial name available, Biomin GmbH, San Antonio, TX, USA) contained the clay mineral diatomaceous earth and yeast culture material. This live yeast culture (*Saccharomyces cerevisiae*) consists of a minimum guaranteed analysis for *Saccharomyces cerevisiae* of 1.2 × 10^10^ CFU/lb. During the 42 days of diet administration, all pigs had free access to feed and water. Concentrations of essential nutrients met or exceeded requirements suggested by NRC (1998). During the 42 days, ADG and ADFI were measured. A protocol for the use of animals was approved by the North Carolina State University Animal Care and Use Committee.

**Table 9:** Composition of experimental diets (as-fed basis).

	Treatment ^1^	
	CON	MT
Ingredient, %		
Ground yellow corn	69.42	4.42
Ground barley	3.00	0.00
Ground yellow corn with aflatoxin ^2^	0.00	65.00
Ground barley with deoxynivalenol ^2^	0.00	3.00
Soybean meal, dehulled	25.00	25.00
Salt	0.30	0.30
Vitamin premix ^3^	0.03	0.03
Trace mineral premix ^3^	0.15	0.15
Dicalcium phosphate	0.90	0.90
Ground limestone	0.70	0.70
Poultry fat	0.50	0.50
Calculated composition		
Dry matter, %	89.50	89.50
ME, Mcal/kg	3.36	3.36
Crude protein, %	18.08	18.08
Lys, %	0.95	0.95
Cys + Met, %	0.61	0.61
Trp, %	0.21	0.21
Thr, %	0.68	0.68
Calcium, %	0.61	0.61
Available phosphorus, %	0.23	0.23
Analyzed composition		
DM, %	88.55	89.97
CP, %	18.71	19.34
Aflatoxin ^4^, µg/kg	<20	150
Deoxynivalenol ^4^, µg/kg	<500	1100
Fumonisin ^4^, µg/kg	<2000	3000
Zearalenone ^4^, µg/kg	<500	<500

^1^ CON: uncontaminated control; MT: contaminated with 150 µg/kg AF and 1100 µg/kg DON; A: MT + 2 mg/kg of a clay additive; B: MT + 1.5 mg/kg of a clay and dried yeast additive; C: MT + 1.1 mg/kg of a clay and yeast culture additive.; ^2^ Corn contained 270 µg/kg aflatoxin; Barley contained 30,000 µg/kg deoxynivalenol; ^3^ The vitamin and trace mineral premixes provided the following per kg of complete diet: 6613.8 IU vitamin A as vitamin A acetate; 992.0 IU vitamin D_3_; 19.8 IU vitamin E; 2.64 mg vitamin K as menadione sodium bisulfate; 0.03 mg vitamin B_12_; 4.63 mg riboflavin; 18.52 mg D-pantothenic acid as calcium pantothenate; 24.96 mg niacin; 0.07 mg biotin; 4.0 mg Mn as manganous oxide; 165 mg Fe as ferrous sulfate; 165 mg Zn as zinc sulfate; 16.5 mg Cu as copper sulfate; 0.30 mg I as ethylenediamine dihydroiodide; 0.30 mg Se as sodium selenite; ^4^ Dietary mycotoxin concentrations were analyzed by North Dakota State University Veterinary Diagnostic Laboratory (Fargo, ND, USA) based on an average of duplicates of each treatment. The quantitation limit is 20 µg/kg for AF, 500 µg/kg for DON and ZEA and 2000 µg/kg for fumonisin.

### 4.2. Blood Sampling

Blood samples were collected aseptically from the jugular vein of one pig per pen (initial median body weight pig) on days 28 and 42. Blood samples were used for hematological, biochemical and immunological analysis. Blood was collected in Monovette tubes (Sarstedt, Newton, NC, USA) containing ethylenediaminetetraacetic acid (EDTA) for hematological analysis. Tubes without anticoagulant were used to collect serum for measuring liver biochemistry, immunoglobulin and cytokine concentrations. Serum samples were allowed to clot at 4 °C before centrifuging for 15 min at 3000 × *g* (4 °C), and were finally stored at −80 °C until analyzed. 

### 4.3. Hematological Measurements

Whole blood with EDTA was sent to Antech Diagnostics (Cary, NC, USA) for complete blood count. Measurements included hematocrit, hemoglobin, mean corpuscular hemoglobin (MCH), mean corpuscular hemoglobin concentration (MCHC), mean corpuscular volume (MCV), platelet number, red blood cell (RBC) count, white blood cell (WBC) count, basophils, eosinophils, lymphocytes, monocytes and neutrophils. 

### 4.4. Immune Parameters

Total concentrations of the immunoglobulin subsets, IgG and IgM, were measured via ELISA, as described by the manufacturer (Bethyl, Montgomery, TX, USA). Goat anti-pig IgG or goat anti-pig IgM were used as capture antibodies to coat wells. Serum samples from one pig per pen were diluted to 1:140,000 and 1:20,000 for IgG and IgM, respectively, and ELISA analysis was completed in duplicate. Horseradish peroxidase labeled goat anti-pig IgG or IgM was used as the detection antibody in combination with the TMB (3,3',5,5'-tetramethylbenzidene) enzyme substrate. A stop solution of 0.18 M sulfuric acid (H_2_SO_4_) was used to stop the enzyme-substrate reaction. Absorbance was read at 450 nm using an ELISA plate reader (Synergy HT, Bio-tek instruments) and KC4 data analysis software. Samples were quantified relative to a standard curve constructed with known amounts of pig immunoglobulin subset. Detection limits were 7.8 to 500 ng/mL for IgG, and 15.6 to 1000 ng/mL for IgM. 

Serum TNFα was measured by ELISA following the manufacturer’s procedure (R&D Systems, Minneapolis, MN, USA). A total of 50 µL assay dilutent RD1-63 was added to microplate wells coated with a monoclonal antibody specific to porcine TNFα, followed by 50 µL of standard, control or sample. Serum samples from one pig per pen were analyzed. Detection occurred by the use of a color reagent substrate and a stop solution of diluted hydrochloric acid. Absorbance was read at 450 nm and 540 nm by an ELISA plate reader and KC4 data analysis software. The detection limit range for TNFα is 2.8 to 5.0 pg/mL. 

### 4.5. Biochemical Serum Assays

Concentrations of alanine aminotransferase, albumin, alkaline phosphatase, aspartate aminotransferase, bilirubin, BUN to creatinine ratio (BUN:creatinine), calcium, chloride, cholesterol, creatinine, creatine phosphokinase (CPK), globulin, glucose, nitrogen, phosphorus, potassium, protein, sodium and sodium to potassium ration were measured (Antech Diagnostics, Cary, NC, USA) for determination of liver function.

### 4.6. Histological Measurements

On day 42, the median initial body weight pig from each pen was euthanized via captive bolt to collect liver, kidney and spleen tissues for weight, color and damage evaluation. Tissue color was measured from 3 locations on each tissue via a Minolta Colorimeter (Konica Minolta, Ramsey, NJ, USA), which measured values of lightness (L*), redness (a*) and yellowness (b*). Samples from the liver and kidneys were fixed in 10% buffered formalin and sent to the North Carolina State University Histopathology Laboratory (College of Veterinary Medicine, Raleigh, NC, USA) for hematoxylin and eosin (H & E) staining and observation of tissue damage. Liver damage measurement included bile ductule hyperplasia, fibrosis, hydropic degeneration, inflammation, karyomegaly, necrosis and vacuolation. Kidney damage measurement included fibrosis, inflammation, necrosis, protein casts, regeneration and vacuolation. Microscopic examinations of tissue damage were measured by an evaluator blinded to treatment, based on the degree of change observed with values of 1: normal to minimal damage (0% to 5%); 2: mild (5% to 15%); 3: moderate (15% to 40%); 4: severe (higher than 40%).

### 4.7. Statistical Analysis

Data were analyzed using the PROC MIXED procedures of SAS (SAS Inst., Inc., Cary, NC, USA), following a completely randomized block design using initial BW groups as a block. Pen was considered as the experimental unit. Separation of means was completed using the LSMEANS of SAS. Treatment effects were considered statistically significant when *p*-values were less than 0.05 and trends when *p*-values were less than 0.10 and equal or greater than 0.05.

## 5. Conclusions

Collectively, the results of our study show that feeding pigs 150 µg/kg AF and 1100 µg/kg DON did impact growth performance, where ADG was reduced by 10.8%, caused some alterations to the immune system and resulted in damage to internal organs. Thus, AF and DON have whole body effects on pigs, even when consuming moderate concentrations of contaminating grains. The addition of feed additives may be beneficial at reducing mycotoxin effects. Although further research may still be needed to understand mechanisms of action, the results of the current study do indicate that both clay and yeast additives can play a role in maintaining pig health under AF and DON challenge. When growth performance was reduced, the three additives numerically increased ADG by up to 6%. When immune functions were altered by the mycotoxins, both clay and yeast materials helped to minimize immune and inflammatory challenges, as measured by the immune parameters IgG and IgM. Another result of mycotoxin consumption was liver damage. In the current study, all three feed additives played a role in minimizing mycotoxin effects on pigs. However, the montmorillonite clay in diet A appeared to provide the most consistent benefits for minimizing mycotoxin effects on the immune system, whereas the additive in diet B, composed of sodium bentonite, sepiolite and dried yeast, more constantly reduced tissue damages. 

Overall, it is concluded that AF and DON can be harmful to pigs when chronically consumed at moderate concentrations of contamination. It is also concluded that the selected feed additives can lessen the negative effects of AF and DON on the immune system and internal organs, subsequently improving the health of the pig, but that the benefits of these additives are variable, and the results are not clear as to which materials provide a stronger protection from the mycotoxins.
